# Climate Change, Ecological Modernization, and Disaster Management: The Coastal Embankment Project in Southwestern Bangladesh

**DOI:** 10.3390/ijerph20126086

**Published:** 2023-06-08

**Authors:** Shaikh Mohammad Kais, Md Saidul Islam

**Affiliations:** 1 Department of Sociology, University of Rajshahi, Rajshahi 6205, Bangladesh; smkais@ru.ac.bd; 2 Division of Sociology, School of Social Sciences, Nanyang Technological University, Singapore 639818, Singapore

**Keywords:** ecological modernization, climate crisis, space for feasible action, shrimp aquaculture, coastal embankment project

## Abstract

Climate change, one of the severest environmental threats to humankind, disproportionately affects low-income, developing countries in the Global South. Having no feasible mitigation alternatives, these countries resort to adaptation efforts to address climate perturbations. Climate change adaptation (or resilience) is primarily a localized course of action that depends on individuals, social networks, economies, ecologies, political structures, and the capabilities of all those to work collectively to absorb, learn from, and transform in the face of new realities. With a view to controlling the floods that shattered the life and economy of the then East Pakistan, which is now Bangladesh, during the mid-twentieth century, the coastal embankment project (CEP) was instituted as an adaptation strategy to natural disasters in Southwestern Bangladesh. Based on a qualitative analysis of primary and secondary data, this paper seeks to critically evaluate the efficacy of the CEP in terms of the space for feasible action and ecological modernization. The findings of this research indicate that the CEP has become an unrealistic venture that hinders the growing economic activity of shrimp aquaculture in the area. This paper is expected to contribute to generating further theoretical and empirical discourse on the evaluation of similar development projects around the globe.

## 1. Introduction

Climate change is, and will remain, the biggest environmental threat in many parts of the world. Because of the long residence time of CO_2_ and other greenhouse gases in the atmosphere, and because of the thermal inertia of the oceans, the current trend of climate change will prevail in the coming centuries regardless of reductions in greenhouse gas emissions [1,2,3,4]. As a result, in addition to mitigation, climate change adaptation has become a pressing issue over the last couple of decades. Climate change adaptation (or resilience) is primarily a localized course of action that depends on individuals, social networks, economies, ecologies, political structures, and the capabilities of all those to work collectively to absorb, learn from, and transform in the face of new realities [5,6,7,8]. Accordingly, resilience in global aquaculture, as in elsewhere, is primarily an outcome of the joint endeavors of various stakeholders. This paper explores, with case studies from Bangladesh, how the ecological modernization (EM) approach can be applied to achieving the expected goals of adaptation strategies. The coastal embankment project (CEP)—originally developed as a mechanism for disaster management to control floods, cyclones, and saltwater intrusion—has been taken as the subject of this investigation. Since coastal Bangladesh embraced a new agrarian transformation with industrial aquaculture, the second largest foreign exchange earner next to garments, the study of CEP is critically important.

At the beginning, we can note a few crucial points. First, although few attempts have been made to test the EM theory at the micro-level with citizen opinion analysis [9,10], this approach largely deals with macro-level issues regarding how governments, businesses, and organizations react to environmental problems [11,12]. Second, the concept of ‘modernization’ implies advancement in the technosphere [13]. Thus, the existing EM literature suggests the cleaning and greening of production and consumption in the capitalist world. In other words, EM studies have so far been conducted primarily in the context of the Global North. Finally, related to the second point, the EM perspective largely addresses the mitigation aspects of global climate change. Since fossil-fuel-based technological accomplishments and ever-increasing capitalist production and consumption patterns are considered to be responsible for much of anthropogenic climate change [14], and since EM focuses on eco-friendly technological overhauling as a means of addressing climate change [13], the EM approach ultimately becomes primarily concerned with mitigation interventions [15], with very little focus on adaptive options, at least up until now. Drawing on our understandings of the role of the EM approach, in this discussion on the applicability of the EM perspective to the commercial shrimp sector in coastal Bangladesh, we concentrate on macro-level interventions to community resilience dealing with how governmental and non-governmental development/adaptation programs, including the CEP, could be ecologically modernized with an aim to foster resilience in the aquaculture communities. The existing literature, in assessing different embankment projects in Bangladesh, resorted mainly to understanding the technical, economic, or environmental aspects of the projects, e.g., [16,17,18,19,20,21,22]. Those studies largely missed out the crucial matrix of the social and political viability of the projects. The present study, however, by applying the framework of space for feasible action, endeavors to unearth the issues from a holistic perspective, taking scientific, technological, environmental, economic, political, and social dimensions into consideration.

## 2. Conceptual Framework

### 2.1. Ecological Modernization

In order to study the efficacy and sustainability of the resilience strategies adopted by the commercial shrimp industry in Bangladesh, this research employed ecological modernization (EM) theory. In explaining the ecological crises associated with modern capitalist society, proponents of the EM theory believe that capitalism as a system is flexible enough to find solutions to the environmental issues and evolve toward “sustainable capitalism” [23,24]. EM theory, originating as a concept in a debate in the Berlin municipal parliament on 22 January 1982 [25] and through the writings of its founding father Joseph Huber [26,27], supports the basic economic and political foundations of the capitalist modernization project. This perspective differs significantly from neo-Marxian radical environmental theories like deep ecology or ecological Marxism theory. While the radical greens or deep ecologists argue that ecological crisis cannot be overcome unless society breaks away from industrial modernity, the EM theorists advocate for reformation of the capitalist economy through technical and procedural innovation [28] and the restructuring of industries pertaining to ecological requirements [29].

In analyzing modern society, EM theorists like Joseph Huber differentiate between three analytical categories or spheres: the industrial system (technosphere), the life world (sociosphere), and nature (the biosphere) [13]. The domination of the technosphere over the other two is the main cause of the emergence of the major problems in modern capitalism. These problems, which are interpreted by the EM theorists as structural design faults of the industrial system, can be overcome by an ecosocial restructuring of the technosphere. Thus, the point of departure for the EM theory is the industrial character of modernity, not the capitalist and/or bureaucratic characters. In other words, EM theorists question the technological structure of modern society, not the economic or political base. Up until now, we have found two notable variants in the EM theory: techno-corporatist ecological modernization and reflexive ecological modernization [12,30]. The techno-corporatist school of EM views ecological reform as a purely techno-administrative affair, while the reflexive school points to practices of “social learning, cultural politics, and new institutional arrangements” [12]. In short, according to EM theorists, ecological problems associated with modernity are a techno-industrial issue that can be overcome through “modernizing modernity in a sustainable way” [29] by adopting technological restructuring or “green technology” and by resorting to reflexive modernity through something like “green consumerism” [23].

One way of viewing the interconnections between modernity and aquaculture is by considering that modern aquaculture itself is a development activity within contemporary neo-liberal globalization processes. In the late twentieth- and early twenty-first-century world, as part of the neoliberal globalization project, transnational corporations and international donor agencies invested in agriculture and aquaculture sectors in the developing countries with the aim of producing foods for global consumption. In the process, we found two revolutions that enhanced global food production but brought about troubled social and environmental legacies with them. These two revolutions are the Green Revolution in agriculture and the Blue Revolution in aquaculture. Influenced by the success of the Green Revolution in agriculture, neoliberal global governance initiated the restructuring of the global aquaculture sector. Commercial aquaculture farming, including industrial shrimp farming, started in 1970s in the Global South. With financing from international donor agencies, large coastal areas in developing countries were converted into aquaculture farms where new technologies were used. As a result, a phenomenal increase in global aquaculture production occurred over the last few decades, which has been dubbed the Blue Revolution [31]. As a modernization activity, aquaculture in general, and industrial shrimp farming in specific, has some negative implications on the environment. However, those environmental ramifications can be solved, from the perspective of the EM theory, through technological overhauling and a turn towards ‘green aquaculture’. In order to ensure a healthy environment, we do not need to overthrow or put a halt on the whole aquaculture industry or the industrial shrimp cultivation in the name of “radical structural change”, as suggested by neo-Marxist political ecology or treadmill of production theorists [23].

That the very process of capitalist industrial development in the Global North is generating threats to the environment and climate of the entire world is another notable issue on the interactions between global aquaculture and neo-liberal modernity. The resulting climate change variability and extremes are affecting aquaculture, somewhat negatively in various regions of the world. Again, in this scenario, the solution lies in evolving towards a “sustainable capitalism” and “super-industrialization” process through an “ecological switchover of the industrial system” [13,23]. Developing a ‘responsible capitalism’ through the ‘cleaning’ and ‘greening’ of technology, as suggested by EM theory, can mitigate the environmental and climate change evils in the current setting—a proposition that has yet to be seen fully in the global aquaculture.

Similarly, the EM perspective can be a crucial tool for understanding how numerous coastal people around the globe can adapt to a changing climate regime, especially to sea level rise (SLR), salinity intrusion, cyclones, and storm surges. Since new technologies and the hard structural interventions of coastal management come with social and ecological costs, coastal agriculture and aquaculture can sustain and even thrive if local authorities utilize ecologically modernized solutions to recent environmental threats.

### 2.2. Coastal Management

With 84% of the countries of the world having a coastline with the open ocean, inland seas, or both, the global coastline is a huge entity totaling 1,634,701 km [32]. Although the coastal zone plays a crucial role in human habitation, agriculture, aquaculture, fisheries, industries, ecologies, and numerous other ecosystem services, the precise conceptualization of the coast is a difficult task. The coast has been variously defined as “the land near a shore” [33], an “area where aquatic and terrestrial ecosystems interact” [34], or “that part of land most affected by its proximity to the sea, and that part of the ocean most affected by its proximity to the land” [35]. In order to cover most of the interactions between the contrasting aquatic and terrestrial ecosystems that co-occur at the coast, Martinez et al. (2007) defines coastal regions as “intertidal and subtidal areas on and above the continental shelf (to a depth of 200 m); areas routinely inundated by saltwater; and adjacent land, within 100 km from the shoreline” [32]. In Bangladesh, three indicators define the coastal region: tidal fluctuation, salinity intrusion, and the risk of cyclones and storm surges (see below “Study area: coastal Bangladesh”).

In terms of natural ecosystems within the 100 km inland boundary, forests comprise 44% of global natural coastal vegetation, shrubs 28%, savannas 21%, and grasslands 7% [32]. On tropical coasts, such as in Bangladesh, mangroves make up a critical portion of coastal vegetation. Globally, mangrove forests cover an area of 14,650,000 ha of coastline [36] over 121 countries [37], with an economic value of about 200,000–900,000 USD/km^2^ [38].

The economic value of the coastal zone lies in the fact that, globally, coastal ecosystems provide numerous goods and services, including food for humans and animals, salt, minerals and oil resources, construction materials (sand, rock, lime, and wood), and biodiversity, including the genetic stock that has potential application for biotechnology and medicine [32]. Additionally, as part of coastal terrestrial ecosystems, mangroves provide benefits under all the four categories of ecosystem services—regulating, provisioning, cultural, and supporting—as defined by the 2005 Millennium Ecosystem Assessment [38]. Regulating services include coastline protection from natural hazards; soil and beach erosion regulation; land stabilization; climate regulation, e.g., carbon sequestration; and water quality maintenance. Provisioning services include subsistence and commercial fishery aquaculture, medicinal products, building materials, fuel wood, and ornaments, e.g., jewelry, decoration. Cultural services include tourism; recreation; spiritual services, i.e., sacred and heritage sites; and aesthetic appreciation. Supporting services include nutrient recycling, nursery habitats, and biodiversity [38,39].

Similarly, the social importance of the coastal zone is enormous. The coast is like a magnet that attracts the world human population by virtue of its beauty, accessibility, and services. People frequent the coasts, for a place to live or for tourism, recreation, leisure, livelihood, and commercial activities. As a result, although coastal areas account for only 20% of all land area in the world, they provide housing for 41% of the world population [32]. This trend, in turn, puts pressure on coastal ecosystems through increasing demand for infrastructural development and economic activities required for the additional population. This pressure may rise in the coming decades since an increasing trend of population movement toward coastal zones is visible. Furthermore, in the warmer world of the near future, it is projected that population pressure will further increase in the coasts, especially in the Global South.

Since coastal areas are among the most inhabited and exploited regions globally, due to their traditionally accepted attractiveness for human living, leisure, and tourism [32,40,41] that leads to the coastalization of people [42], they are always a source of dispute among various stakeholders who engage in conflicting coastal land uses (user–user conflicts) and between human activities and the environment (user–environment conflicts) [43,44,45,46]. Coastal authorities throughout the world implement new interventions in coastal areas in order to address both types of conflicts and the natural changes caused by new environmental and climate regime and to further social and economic development. Nonetheless, the execution of a development or protection project in a coastal zone is a multifaceted issue. The conflicting interests of the stakeholders and the ever-changing nature of the coastal ecosystem often cause intervention schemes, such as the CEP in Bangladesh, to generate a series of reactions [42]. In the Bangladeshi context, the competing and conflicting land use patterns on the coast that cause crucial concerns to the central and local administrations include agriculture, aquaculture (especially shrimping), fisheries, salt production, forestry, shipbreaking yards, ports, industry, tourism, human habitation, wetlands, and recreational activities [47]. Although embankments are viewed as a human intervention for addressing natural perturbations, embankments are a kind of land use that impact other types of land use in the region. Consequently, sometimes conflicts of interest surrounding the projects arise among different stakeholders.

## 3. Materials and Methods

The aim of this study was to explore the applicability of the ecological modernization framework in assessing the efficacy of government-run adaptation schemes in coastal aquaculture communities. In other words, we (the authors) try to appraise the CEP in Bangladesh, taking it as a case study, in light of the space for feasible action. In doing so, we have focused on collecting in-depth qualitative data from various stakeholders so that a critical understanding can be achieved. A triangulation of methods, involving content analysis of secondary sources, ethnography, and in-depth interviews, was applied in order to acquire a comprehensive insight into the complex and diverse issues of climate threats, economic activities, and the feasibility of the CEP in the research area.

### 3.1. Study Area: Coastal Bangladesh

The Bangladesh landmass is connected to the Bay of Bengal in the South via a 710 km long coastline [48]. The coast is not a fixed, static line between land and sea; rather, it is intersected by a vast network of river systems, an ever-dynamic estuary, and a drainage basin, through which a huge amount of freshwater is discharged into the sea from Bangladesh and parts of India, Nepal, Bhutan, and China. The Coastal Development Strategy of 2006 employed three physical criteria in defining the coastal area in Bangladesh: the limits of tidal fluctuation (difference of 0.3 m between the high and low tide in a day), salinity intrusion (4 dS/m, 5 dS/m, and 2 dS/m as the threshold salinity levels for soil, surface water, and ground water, respectively), and the risk of cyclones and storm surges (among the high risk, risk, wind risk, and no risk zones mapped by the Disaster Management Bureau, the first two indicate coastal areas) [48,49].

**Figure 1:**
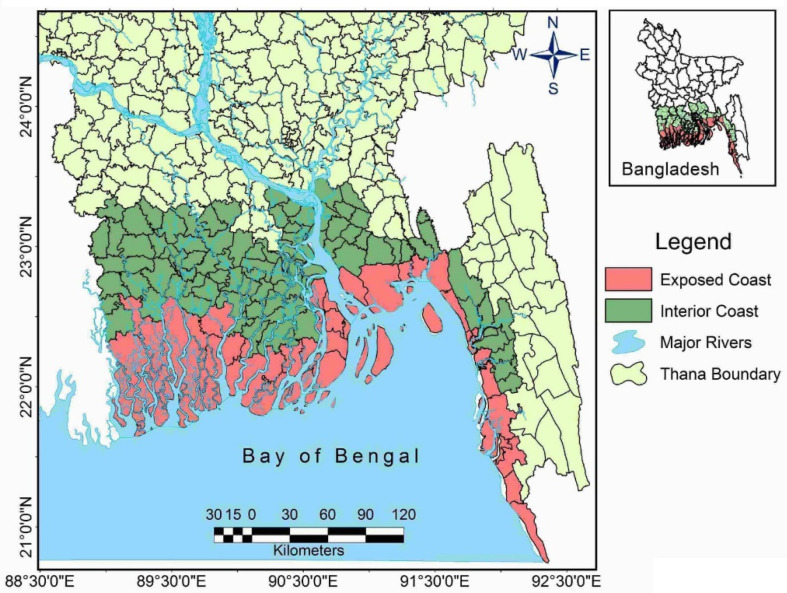
Map of the coastal region of Bangladesh (Credit: Md Samsul Kalam).

An upazila (sub-district) is considered to be ‘coastal’ (Figure 1) if at least one of the defining parameters of a coastal area is at the threshold level in the upazila. A district is termed as ‘coastal’ if it includes at least one coastal upazila. In Bangladesh, 133 upazilas under 19 districts (Bagerhat, Barguna, Barisal, Bhola, Chandpur, Chittagong, Cox’s Bazar, Feni, Gopalganj, Jessore, Jhalkathi, Khulna, Lakshmipur, Narail, Noakhali, Patuakhali, Pirojpur, Satkhira and Shariatpur) are defined as coastal. In 48 upazilas across 11 districts, all three indicators are above threshold level, making them “exposed coastal zones” [48], while the remaining area is referred to as the “interior coast” [50]. The exposed coastal districts were defined as the “southern region of Bangladesh” in a Master Plan of the Bangladesh Government [51]. In total, the coastal zone covers an area of 47,201 km^2^, 32% of the total landmass of Bangladesh [50] and is home to 46 million people [52].

**Figure 2:**
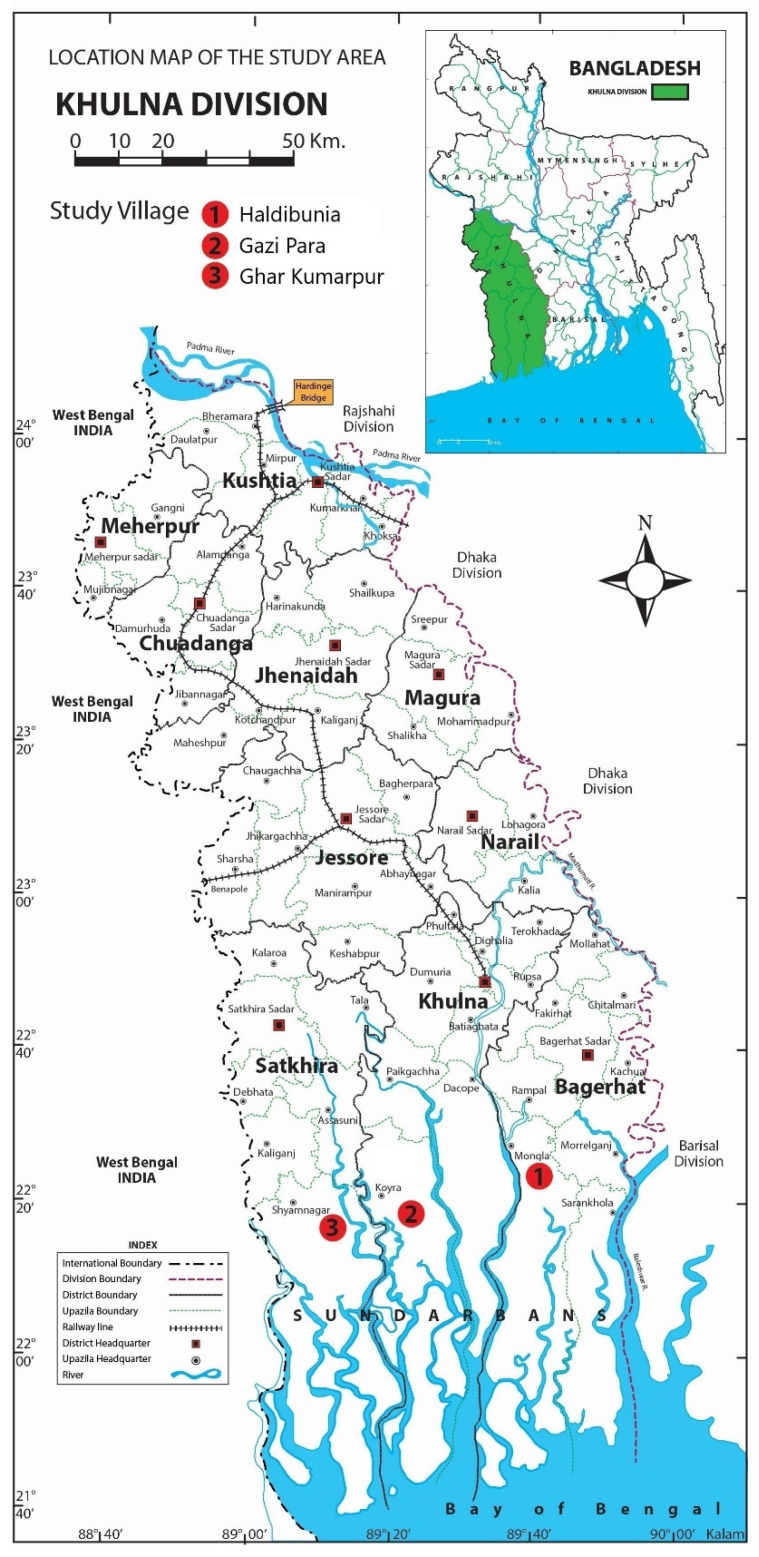
Map of the research areas (Credit: Md Samsul Kalam).

For this study, we selected three upazilas (sub-districts): Mongla from the Bagerhat district, Koyra from Khulna, and Shyamnagar from Satkhira. For close investigation, we again picked three villages from these three sub-districts: namely, Haldibunia (Mongla), Gazi Para (Koyra), and Ghar Kumarpur (Shyamnagar) (Figure 2). All of these upazilas are situated in exposed coastal regions. The vast majority of the inhabitants of the selected villages are engaged in shrimp farming and shrimp-related activities. According to the Bangladesh Bureau of Statistics (BBS), 79.43% of employed males in Haldibunia in Mongla [53] 95.65% in Gazi Para in Koyra [54], and 80.67% in Ghar Kumarpur in Shyamnagar [55] are engaged in agriculture (BBS defined shrimp cultivation as an agricultural activity). The study areas are vulnerable to anthropogenic climate extremes including SLR, salinity ingress, cyclones, and storm surges. There is a total of 227 km paka (carpeting) road across the three upazilas, and 275 km of brick-soling road, out of more than 1737 km of road. The total length of the BWDB embankment in Koyra and Shyamnagar is 339 km with 46 sluice gates (information collected from Upazila Nirbahi offices). Although there is no embankment in Haldibunia, Mongla, we took this site for our research in order to understand people’s experience of exposure to climatic events in an unprotected area. This enabled us to appraise the efficacy of the CEP in a holistic manner.

### 3.2. Content Analysis

At first, to gain a thorough understanding of the CEP and the applicability of the EM theory to it, we (the authors) resorted to a content analysis of the existing secondary documents in Bangladesh. After mapping out the entire project, we searched libraries of various institutions and organizations in Dhaka, Khulna, Satkhira, and Bagerhat, including District Fisheries Offices, Bangladesh Water Development Board (BWDB) offices, the Bangladesh Centre for Advanced Studies (BCAS), the Bangladesh Unnayan Parishad (BUP), the Bangladesh Disaster Preparedness Centre (BPDC), the Centre for Environment and Geographic Information Services (CEGIS), the Climate Change Cell of the Department of Environment (DoE), the Department of Fisheries (DoF) under the Ministry of Fisheries and Livestock, the Coastal Development Partnership (CDP), the Bangladesh Institute of Development Studies (BIDS), the Bangladesh Frozen Foods Exporters Association (BFFEA), the World Bank Country Office, the Bangladesh Bureau of Statistics (BBS), the Institute of Disaster Management and Vulnerability Studies (IDMVS) at the University of Dhaka, the Shrimp Research Station of the Bangladesh Fisheries Research Institute (BFRI), etc. From these sources, we collected robust information on various topics related to our research, such as the history and present condition of the CEP, the environmental and ecological issues of the area, the nature of existing conflicts surrounding resource use in the coastal area, legal-political issues of shrimping, local and national climate change dynamics, etc. This phase of content analysis helped us to familiarize ourselves with the whole set up of climate change vulnerabilities, adaptation policies and programs, and embankment projects that are currently ongoing or planned for future realization in Bangladesh.

### 3.3. Ethnography

In the next phase, we arranged three ethnographic visits to three coastal districts in which, along with other coastal districts, the CEP was instituted—Bagerhat, Khulna, and Satkhira. As a data collection method, ethnography involves the researcher “participating, overtly or covertly, in people’s daily lives for an extended period of time, watching what happens, listening to what is said, and/or asking questions through informal and formal interviews” [56]. Ethnographic inquiry involves a “prolonged, systematic, first-hand and direct encounter” [57] with the people concerned in their own cultural “settings” [58] through their lived experiences [59]. The main aim of the ethnographer is to provide a “thick description” [60,61,62] as well as an “interpretation” [57] of the observed pattern of human activities [63]. The ethnographic visits to the research areas helped the researchers understand the on-the-ground scenario regarding the pros and cons of the CEP.

### 3.4. In-Depth Interview

At the final stage, after obtaining a clear insight into the local dynamics through ethnography, we interviewed selected people from different stakeholder groups. They included three upazila agricultural officers, three upazila fishery officers, three BWDB officials, three other key informants (experts), and fifteen shrimp cultivators (Table 1). In selecting the respondents, we resorted to the purposive sampling technique, which allowed us to find the most useful or representative samples. We used an unstructured interview schedule in order to collect in-depth qualitative data from them. As part of our research design, we talked to the participants for extended periods of time on various issues, including the effect of the CEP on the respondents’ economic activities, conflicts surrounding divergent land use in the locality, their views on the environmental consequences of the embankment, their understanding of the changes in local climate, and so on. Taken as a whole, we took exhaustive efforts to understand the local dynamics in relation to this research.

## 4. Results and Discussion

### 4.1. Ecological Modernization Approach to Addressing Adaptation and Resilience

Ecological modernization, as a broad environmental paradigm, encapsulates different versions—from a set of attractive policy ideas that are not real [30] to an identifiable phenomenon of institutional reflexivity [26]; from mild corrective activities to radical movements that re-cast the role of the state [15,64]. As Hajer (1995), Weale (1992), and others have described, ecological modernization has succeeded in reducing pollution, cutting down wasteful resource use, and addressing other environmental issues throughout the Global North [30,65]. EM can play a role in channelizing resilience and adaptive actions in the Global South in a way that prevents or minimizes perverse consequences, restricts the rise of “maladaptation” (see [66,67,68] for discussion on maladaptation) and promotes “sustainable adaptation” [3,69,70,71,72,73,74].

Up until recently, adaptation was thought to be benign for development; thus, the negative social and environmental consequences of coping mechanisms were long overlooked. However, balancing trade-offs and avoiding potential negative outcomes have now, especially after a double session on sustainable adaptation at the Human Security in an Era of Global Change Conference in June 2009 [71], become a growing concern for adaptation and resilience schemes throughout climate challenged societies. A few researchers have already explored the negative implications of planned and autonomous adaptations in developing countries [75,76,77]. The spatial and temporal negative consequences of an adaptation action may turn it to become a maladaptation. Maladaptation can be defined as “action taken ostensibly to avoid or reduce vulnerability to climate change that impacts adversely on, or increases the vulnerability of, other systems, sectors, or social groups” [67], or “a result of an intentional adaptation policy or measure directly increasing vulnerability for the targeted and/or external actor(s), and/or eroding preconditions for sustainable development by indirectly increasing society’s vulnerability” [68:139]. Thus, adaptation can become maladaptation if it creates increased exposure and sensitivity for the actors, targeted actors, or others and if it decreases the adaptive capacity of the actors, targeted actors, or others [68]. We can cite a few examples of maladaptation from around the globe: (a) increased exposure: trees planted in Scandinavian countries to provide shade, which damage buildings during a storm [78]; (b) increased sensitivity: development of floodplains that leads to reduced buffering capacity of river water [79]; and (c) decreased adaptive capacity: investments in power grids in Australia leads to increased prices of power, decreasing the adaptive capacity of vulnerable groups [80].

In order to avoid such maladaptive policies or strategies that bring positive outcomes to people of a particular time and space but may have negative effects on some other groups in other areas or at other times, it is crucial to identify the synergies between adaptation and sustainable development and devise schemes of ‘sustainable adaptation’, i.e., “adaptation that contributes to socially and environmentally sustainable development pathways” [3]. A synergy can be established between sustainable adaptation and ecological modernization, in which the basic features of EM can foster sustainability in adaptation (resilience) policies and actions. Glover identified a few aspects of EM that can serve as foundations for adaptive strategies in responding to climate change: (1) “actions will reduce the costs of climate change impacts for current and future generations”, (2) “adaptation will protect, secure, and promote future economic growth”, (3) “adaptation actions will serve as a source of innovation”, and (4) adaptation actions should focus simultaneously on scientific information and advice, as well as on institutional frameworks and processes [15].

In order to serve the above functions in respect to resilience initiatives, EM places social learning at its heart and focuses on the societal need for detecting and understanding new climate issues, for framing such issues in ways with which different powerful actors comply, and for responding to such issues in ways that are technologically, economically, and politically viable [81]. In this way, EM promotes the building of a “space for feasible action” [81,82] in society, through which climate challenges and other environmental problems can be addressed within the existing social context. Space for feasible action on climate change lies in the intersection of policies and programs that are “scientifically justified, technologically possible, economically viable, socially supported and politically accepted” [81].

**Figure 3:**
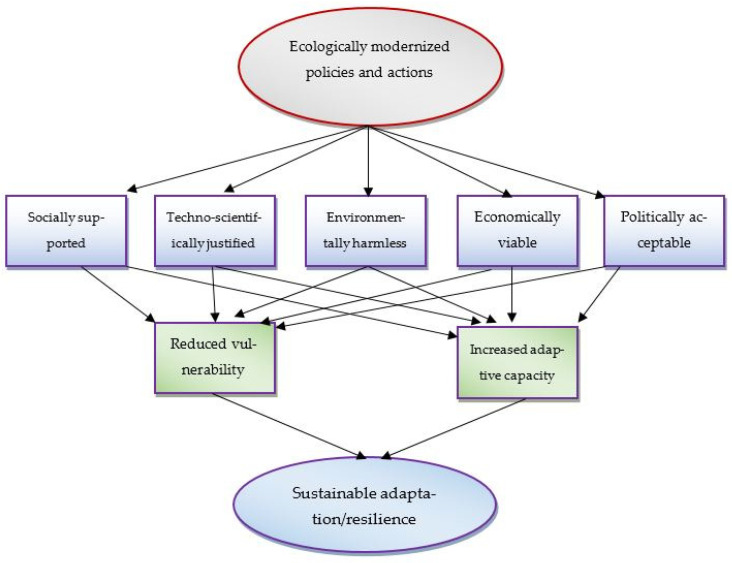
Ecological modernization pathways to sustainable adaptation.

We can add ‘environmentally harmless’ as the sixth component with the above five. If one or more of these pre-conditions are not fulfilled, the space for feasible action can be restricted, and hence, the progress of the actions addressing climate change may be hampered. Thus, for any adaptive or resilience action to be fully sustainable in the long run, it should meet all the criteria of a feasible action. If any program fulfils one or more criteria but not all, it may be feasible to some degree or for the time being. Ecological modernization, thus, by creating space for feasible resilience actions, promotes sustainable adaptation, which reduces vulnerability and increases the adaptive capacity of the actors, targeted actors, or others. Figure 3 shows the ecological modernization pathways to sustainable adaptation.

### 4.2. The Coastal Embankment Project in Bangladesh: A Case Study

We can apply this framework to judging resilience policies and programs designed for the aquaculture industry in the Global South. Here, we will assess a case study from coastal Bangladesh, which will explain how an adaptation effort (in this case, the coastal embankment project) can turn out to be maladaptive if it is not designed as an ecologically modernized action and does not meet the criteria of spaces for feasible action.

Though vernacular embankments have always been a part of the cultural landscape in the coastal regions of Bangladesh [20,83], systematic development of large-scale embankments for flood control started in the 1960s [84,85,86,87,88]. After Bangladesh (then East Pakistan) experienced severe floods for three successive years in 1954, 1955, and 1956, the UN, following a request from the Pakistani Government, sent a technical assistance mission in 1957 under the leadership of J. A. Krug [86,87]. The Krug Mission made a number of recommendations that eventually laid the foundation of flood control policies in Bangladesh. The coastal embankment project (CEP) in East Pakistan was initiated as per one of those recommendations and as part of a master plan for water and power development [89]. The newly formed East Pakistan Water and Power Development Authority (EP-WAPDA, established in 1959) undertook this gigantic project as a hard, structural response to the existing environmental problems in the region [90].

**Figure 4:**
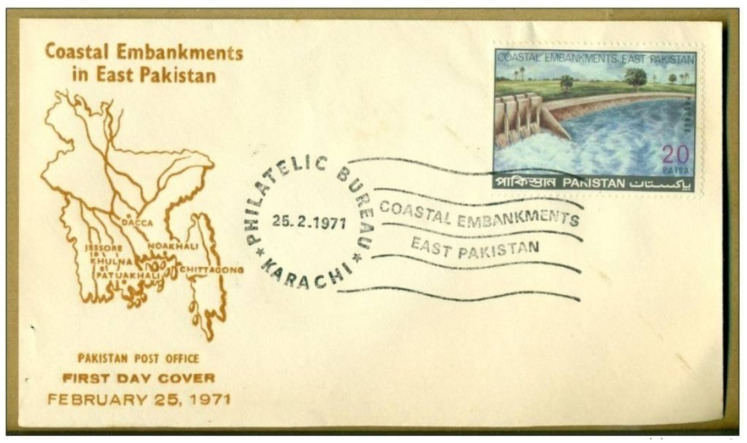
First day cover of a postage stamp of Pakistan on the Coastal Embankments in East Pakistan, 1971.

With an intended 350,000,000 cu yd of earth being moved [85], the project was believed to be one of the biggest earth moving jobs in the world at that time. The CEP, which covers all the exposed coastal districts of Bangladesh, comprises a complex network of dikes and drainage sluices (see Figure 4) and was the first extensive plan for providing protection against floods and salinity ingress in the coastal region. The project, completed between 1961 and 1978, created 139 polders (embanked areas) [51,91], 5017 km of embankment [88], and 1039 drainage sluices [84], and protected around 1.5 million hectares of land [88].

The CEP was designed with a view to ensuring the food security of the people of the country by increasing the yield of agricultural crops in the coastal reclaimed land through protecting the area from tidal and storm surge flooding and saltwater intrusion. It was estimated that the CEP would result in an increased rice production of 480,000 tonnes per year [85]. Initially, this project was successful in achieving its target, as demonstrated by the increased rice production in the area for few years [51].

However, as time passed, some serious ecological and social problems, termed “second generation problems” [51], emerged in the project area as a result of impoldering. First, this huge anthropogenic activity—along with other anthropogenic interventions like the reclamation of forestland, deforestation, agriculture, and the construction of upstream dams—caused changes in the natural processes of land formation and altered the topographic and hydrologic landscape of the area [5]. The floodplain delta is still under active formation through natural processes, including regular shifts in the courses of rivers and the emergence and submergence of coastal islands. Sediment accumulation from rivers meets with the coastal forces of tides, cyclones, and storm surges to form the fluid landmass through a constant process of erosion and accretion [5,92]. The artificial changes in the natural landscape brought by the embankments while the delta was still in a state of immaturity created a division among the land users in the coastal areas—polders and unprotected areas [93]. The polder areas were no longer subject to the regular flushing of tidal water, and hence, land formation through natural siltation was severely hampered.

**Figure 5:**
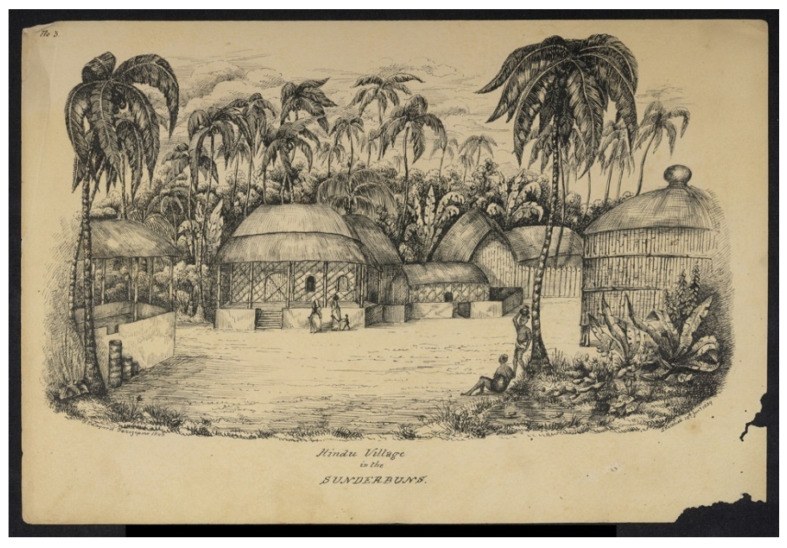
A village in a reclaimed area of the Sundarbans. Drawing by Frederic Peter Layard following an original sketch from 1839. Source: British Library (2016) [95].

Second, the CEP resulted in shifts in the livelihoods of a portion of people who had previously depending on fishing in the beels—i.e., small, depressed areas. Historically, the Sundarbans area was a land of fishers. Early settlers were completely dependent on water resources for their livelihoods. Human settlement in the Sundarbans area dates back to almost two thousand years ago. The ruins of a city, which was located near the present-day Baghmara Forest Block in West Bengal, indicate that it was presumably built by Chand Sadagar, an elite Indian merchant, around AD 200–300 [94]. Historical records and artefacts also suggest that there had been a flourishing agrarian settlement in the region for centuries before the Mughal invasion of Bengal in 1128. Later, Indo-Turkish Muslims continually settled in the Sundarbans area from 1204 to 1574 [94]. This was the period when the indigenous inhabitants had to adopt new livelihood options. The early Hindu settlers were fishers for centuries, but the Muslims introduced a cultural penchant for crop agriculture. Figure 5 portrays a village in a reclaimed area of the Sundarbans.

During the Middle Ages, the whole area of the Sundarbans was depopulated significantly; several reasons have been forwarded to explain this, including earthquakes and natural calamities that led to a sudden subsidence of the land [96,97], attacks by Portuguese and Arakanese pirates [98], and a hostile environment [99,100,101]. Thus, before the 19th century, the Sundarbans were very sparsely settled. Human settlements in the region again soared under British agrarian and land tenure policies [94,98,100] that focused on the generation of revenue by using and managing the forest and its resources in new ways. The effects of these measures still affect the area’s present-day ecology, landscape, land use, social organization and livelihoods. In the pre-colonial period, there were instances of reclamation of the Sundarbans land, but those efforts were mainly individual, isolated, and erratic in nature and did not leave any long-lasting marker on the physical and geographical landscapes of the region. Only the British policy pushed the jungle back significantly through clearance and occupation. The British passion for economic gains through the commercialization of agriculture ramped up the amount of forest converted to rice cultivation markedly. British Collector General Claude Russell in 1771 [100], and later in 1783 the then Magistrate of Jessore, Tilmann Henckell [98], set off an arrangement to divide the Sundarbans into plots and to lease them out to local zaminders (landlords) for timber extraction, land reclamation, and the collection of revenues. These zaminders engaged poor people from different parts of Bengal in clearing and developing land for crop agriculture. Thus, it was agriculture, not aquaculture or shrimp cultivation, which destroyed the mangrove forest of the upper regions.

**Figure 6:**
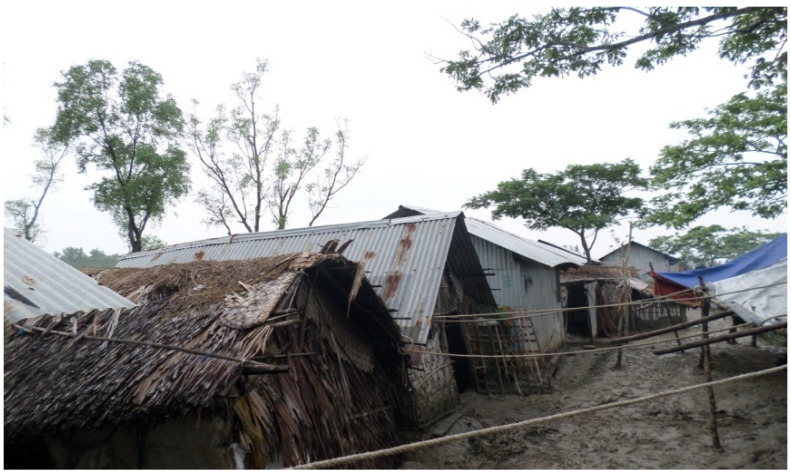
A Bagdi settlement on the riverside of an embankment in Koyra. Source: Field data.

Even after the above-mentioned land reclamation initiatives, the economy of the region was primarily based on fishing because of the abundance of natural water resources in the area. Crop agriculture was the secondary profession for only a few months in a year [20], when freshwater was available. Unlike in many parts of the country, farmers in the coastal zone can produce only one agricultural crop in a year, and not three. Thus, the CEP was an initiative that aimed at forceful conversion of a saline water ecosystem into a freshwater zone. Moreover, after this conversion, as reported by the locals, the fishing community, locally known as Bagdi, was affected badly since the availability of brackish-water fish species declined in the beels in the polders. The Bagdi community (Figure 6), according to members of this community, was forced to depend only on the rivers, and many of them had to change their livelihoods. Though there were other reasons behind this shift—such as a reduction in fish availability in the rivers—the CEP had an effect.

Third, the CEP resulted in the alteration of natural watercourses in the region. As mentioned earlier, earthen embankments are an age-old practice in the coastal region [102], with the earliest record of coastal embankments in Bangladesh dating back to the seventeenth century [84], when the local zaminders patronized the construction of small-sized embankments, and by 1900, Bengal had 1298 miles of embankment [103]. However, those embankments were small in size and were separate from one another, each enclosing relatively small piece of land, and many of them were constructed seasonally for cultivation. Thus, those small-scale embankments did not impede the natural water circulations in the region. Regular flushing of tidal water served in many ways: it helped the formation of land through siltation; it brought nutrients with the silt, which increased fertility of soil; and it provided the local people with a livelihood based on abundant wild fish and other aquatic resources. With the large-scale construction of coastal embankments, the natural flow of water was restricted to outside of the polders, and thus, the impoldered land was devoid of the above-mentioned benefits. This has turned out to be an ecological crisis in the region because of the serious alteration to the natural brackish-water-based ecosystems.

Fourth, related to the third problem, the land levels in impoldered areas gradually dropped with respect to sea level because of subsidence, the settlement of upper layers, climate-induced SLR, and the absence of new sedimentation within the polders. Natural delta systems respond to these phenomena by increasing the rate of sedimentation and accretion, but impoldered areas are isolated from such accretion activities [93]. This has resulted in a number of spillover effects: (a) Because of the siltation of outfall channels, canals and other channels within impoldered areas lost their drainage capability, resulting in waterlogging. This waterlogging problem is exacerbated by the fact that a significant number of sluice gates (nearly half of those in Koyra and Shyamnagar) are currently out of order. Moreover, persistent rainfall during the monsoon also causes water logging because of the shortage of drainage channels. (b) With a height ranging from eight to twenty feet, the embankments are not designed to provide protection against cyclonic storm surges. Moreover, the relative height of the embankments has already reduced because of the elevation of the ground level in rivers through siltation, as mentioned above, and because of anthropogenic SLR. Thus, the embankments have now become more vulnerable to the overtopping of storm surges. Moreover, if saline water from the sea enters the polders in large quantities, water and soil salinity will increase rapidly, sometimes crossing threshold limits even for the old trees. This is evident throughout the Aila-affected areas in the region. After Cyclone Aila in 2009, since many of the areas were inundated with salt water for up to two years because of drainage congestion, the production of vegetables and domestic trees has been quite difficult because of the permanent salinity increase. (c) The above-mentioned issues have led to severe a scarcity of potable water in the Aila-affected areas. In some villages, people need to travel far and spend considerable sums of money and time to fetch drinkable water for their households. If the water scarcity continues for a long time, according to Rahman and Kabir (2013), this may even begin to disintegrate the coastal settlements over time [20].

Fifth, though the embankments are not primarily designed to provide protection from cyclonic surges, people living inside might have a “false sense of security” [20] and build their houses without taking sufficient safety measures, which can make them even more vulnerable to climate disruptions.

Sixth, the CEP also prevented the natural expansion of the mangrove areas. Since the expansion of mangrove forests is a natural phenomenon that occurs through spreading of mangrove seeds through water in nearby areas and throughout the saline water areas, coastal embankments retarded this process, at least to some extent, by restricting saline water outside the polders [20].

Seventh, the CEP reduced the natural habitation of the shrimp fry in the area, which may eventually affect the shrimp industry [20]. Eighth, the CEP also instigated conflicts between different land-users, including shrimp and rice cultivators. Shrimp farmers bring saline water to the polders in various ways, which may affect standing crops in the surrounding plots. There are records of confrontations in the past affecting the social fabric in the area. Multi-functional land use was not in consideration during the construction of the polders, and the BWDB has had no effective strategy to date to address the land use conflicts [51]. Finally, many of the embankments are now under the threat of collapse because of overtopping, toe erosion, slope erosion, and an inadequate operation and maintenance (O and M) budget [88].

### 4.3. Efficacy of the Coastal Embankment Project

From the above case study on the coastal embankment project, we can draw a few conclusions about the impact of the CEP on coastal Bangladesh and its shrimp farming community with respect to space for feasible adaptive action and ecological modernization. The CEP was an adaptive action taken by the central government in response to weather shocks and climate hazards—namely, coastal floods and salinity intrusion—with a view to enhancing the resilience of the coastal communities. Though initially the project was a success in terms of protecting the coastal communities from natural disasters and increasing crop yields, it turned to be maladaptive in the end.

**Figure 7:**
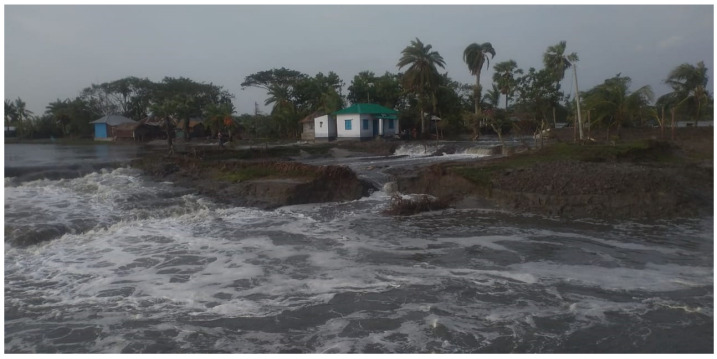
Part of an embankment in Shyamnagar damaged during Cyclone Amphan in 2020. Source: Field data.

First, the CEP was politically acceptable since there was no known case of protest from any quarter of people, including the shrimp culture community, during the initial period of implementation. In this sense, it is a feasible adaptive action. Second, initially, the project was technologically and scientifically justified. The EP-WAPDA appointed Leedshill-De Leuw Engineers as consultants [85] for the project, who completed it successfully over the span of 17 years. All of the components of the project—including the height, width, and slopes of the embankments; drainage sluices with flap gates; and the materials and methods of construction—were techno-scientifically justified and feasible as per their design. However, in the end, the techno-scientific justification did not remain valid. The embankments have become weak because of regular tidal wave action and occasional cyclone-related water surges that exert a tremendous hydrological load on the embankments, resulting in toe erosion and causing damage to the structure [88]. Already, the embankment has been damaged severely in several areas during cyclonic storm surges over the last decade (Figure 7).

Similarly, slope erosion from natural causes such as rainfall, piping action, poor design (e.g., insufficient setback), substandard construction, and insufficient compaction have led to the weakening of the embankments [88]. Thus, though the project seemed to be techno-scientifically justified at the beginning, it eventually turned into an ineffective action, from a technological point of view.

Third, as noted above, this project produced significant environmental and ecological concerns in the coastal areas, affecting both physical setting and agro-aquaculture. The CEP can in no way be treated as an environmentally benign action. Fourth, again, the project was at first economically viable if we consider it from the funding point of view. The total estimated cost of the project was USD 238 million [85], funded by USAID and the Pakistan (and later the Bangladesh) Government. The economic return from rice cultivation was, as noted above, satisfactory in the first few years, up to the 1980s. Initially, the EP-WAPDA estimated an increase in rice production of over 480,000 tonnes per year, with a 2.56:1 benefit–cost ratio for agricultural yield [85]. Nevertheless, within a decade of its completion, the CEP started losing its positive impact on agricultural yield in the polders. As discussed above, the rice production was hampered due to the decreasing fertility of soil because of the stoppage of regular tidal flushing on it. Again, starting in the mid-1980s, the embankments were overtopped by saline storm surges several times, causing long-term water logging and salinity ingress in water and land because of drainage congestion, which also decreased overall rice production. Shrimp farmers in Koyra and Shyamnagar reported that in most areas, shrimp culture started during the 1980s, when the region was flooded by saline water associated with cyclonic storms. Now, after Cyclone Aila, rice production in the polder areas has become completely unfeasible. At present, no serious farmer wants to cultivate rice on his land, since the cultivation of rice in the area is not beneficial economically from a cost–benefit perspective. Moreover, even salinity-resistant rice varieties cannot tolerate the current salinity level in the water and soil of the research areas [104]. Thus, the single most important goal of the CEP—i.e., an increase in freshwater crop yield through preventing salinity intrusion—has completely failed.

There has been no serious study to date on the economic impact of the CEP on shrimp aquaculture. When the CEP started in early 1960s, shrimp cultivation for the market did not spread so much; the industry was in its nascent stage. Shrimp culture had been increasing slowly in the 1970s and made a leap forward in the 1980s and 1990s. Since there is a lack of documentation on the role of coastal embankments, this study tried to gauge it through information from senior inhabitants of the research areas. Local people reported, as mentioned above, that shrimp farming in Koyra and Shyamnagar was sporadic and was concentrated mainly in the ‘unprotected areas’ outside the polders, especially before the mid-1980s. Similarly, in Mongla, though there is no BWDB embankment in the upazila, shrimp farming was a very small-scale business before the 1980s. The growth of the shrimp industry in Bangladesh is connected to the global food regimes. The neoliberal global food governance project, through the restructuring of global aquaculture under the Blue Revolution, “open[ed] up the natural resource pool of the Global South to satisfy the appetites of wealthy consumers in the global North” [31]. Initially, local landowners were not aware of the huge economic return from shrimp farming in the area, and some entrepreneurs from outside started to take the land on a lease from the owners and introduced large-scale shrimp culture in the areas. At the beginning, as discussed above, there were incidents of confrontation between outsider entrepreneurs and local landowners. Thus, when local people became aware of the profitability of shrimp culture, they started cultivating shrimp on their lands. Coastal embankment played both positive and negative roles in the shrimp aquaculture. It restricted the expansion of shrimp culture, since salt-water intrusion in the polders was prohibited initially. On the other hand, after the introduction of shrimp farming in the polders, the embankments provided protective support from natural disasters, at least to some extent.

The economic viability of an embankment also depends on its regular maintenance. The infrastructure is subject to wear and tear by natural and environmental factors, such as erosion, cyclones, storm surges, and floods; by human interventions; and by animal activities. Thus, preventive, periodic, and emergency maintenance, as well as rehabilitation (if necessary), is crucial for the sustainability of the embankment. However, in Bangladesh, a sufficient amount of funding is not allocated for O and M of the BWDB projects. Since 1959, the BWDB implemented at least 710 projects, with an estimated total value of investment of BDT 200 billion. Since annual O and M costs amount to 3% of the total investment costs as a rule of thumb, the yearly requirement for O and M of the BWDB infrastructure is BDT 6 billion [93]. However, the O and M budget of the BWDB never exceeds half of the required amount. Lack of resources has restricted even routine O and M of the embankments, making them weak and fragile [88]. Thus, the economic viability of the CEP, when considered from a space for feasible adaptation perspective, is largely questionable.

Finally, the social acceptance of the embankments—as the embankments are at present—is not at a reasonable level. As mentioned above, after the initiation of large-scale shrimp farming in the polders by the end of the 1980s, land use conflicts between aquaculture and agriculture led to several confrontations in the impoldered areas. Initially, the shrimp cultivators also breached the dams to exchange gher water. There were also some initiatives to restrict shrimp cultivation in the polders during and after the 1990s, but these initiatives brought no results. Since crop agriculture is not feasible at all in the areas, people are compelled to resort to salt-water shrimp farming. Respondents from Koyra reported an event. In 1999, the then MP requested that all farmers restrain from shrimp cultivation in Koyra. Following the appeal of the MP, almost none of the local people cultivated shrimp in the area for two consecutive years. Nevertheless, most of them eventually incurred a loss by cultivating rice. Then, the people of the community arranged a meeting to solve the issue. In that meeting the landowners and the shrimp farmers agreed to restart shrimp farming in the area by bringing salt-water into the polders. This example illustrates the low level of social acceptance of the CEP in the shrimp-farming community.

## 5. Conclusions

After considering all of the above components of spaces for feasible adaptation actions, we can conclude that the CEP, at least in its current state, cannot be referred to as a fully ‘sustainable adaptation’ strategy in addressing salinity, storm surges, floods, or cyclonic phenomena in the coastal shrimp communities of Southwestern Bangladesh. Its role in reducing vulnerability and increasing the adaptive capacity and resilience of the coastal aquaculture and agriculture communities are very much a questionable phenomenon. Realizing the severe ecological, environmental, economic, and social drawbacks of this project, the District Fisheries Officer of Khulna termed it “a development massacre”, the president of the Bangladesh Shrimp and Fish Foundation (BSFF) called it “an assault on nature”, and a shrimp farmer from Mongla dubbed it “a premature action”.

To make the project a space for feasible action, a few ecological modernization schemes need to be incorporated into it. The height of the embankments should be increased to a level that will protect the impoldered area from cyclonic storm surges. A sufficient number of two-way sluice gates should be installed so that these gates can be used for the intrusion of regular tidal water from rivers, the flushing of which can contribute to land formation as well as to the increase in soil fertility through natural siltation within the polders. In addition, these gates could be used for draining extra water during the monsoon and passing river water into shrimp farms when needed. An effective system of drainage channels should be erected to avoid waterlogging in the polders. The regular dredging of rivers along the embankments is required for avoiding siltation outside the polders. Periodic, preventive, emergency, and rehabilitative maintenance should be ensured to obtain optimum benefit from the project. To meet this end, sufficient funds need to be allocated. Furthermore, taking the above-mentioned ecologically modernized initiatives, the polders should be redesigned for multi-functional land use, eradicating any confrontation among the land users. Finally, in order to make the CEP an ecologically modernized project, we recommend that the authorities pay attention to a number of issues, such as tracking and monitoring the long-term social, economic, and environmental effects of the project and evaluating and strengthening the management of the project on a regular basis. From our brief case study of the CEP in coastal Bangladesh, we can conclude that resilience and adaptation schemes in aquaculture can be ecologically modernized to prevent any maladaptive consequences and to establish a sustainable aquaculture practice in the new climate regime.

The potential contribution of this research to the literature on climate change and coastal studies lies in the fact that, as mentioned earlier, this study demonstrates a novel attempt to appraise a government-run intervention project in the Global South in light of the ecological modernization approach. This study applies the EM perspective in evaluating an adaptation program, in contrary to the existing trend of using this perspective in climate change mitigation schemes in the Global North. It is also novel in terms of scrutinizing an embankment project through the lens of space for feasible action. Going beyond the popular techno-economic perspective of project evaluation, this study equally highlights all major dimensions, such as the techno-scientific, environmental, economic, political, and social feasibilities of the CEP. Thus, it is hoped that this holistic approach of mega-project evaluation can be replicated in similar projects around the globe.

Using robust methodologies, this study is a sociological investigation of the CEP focusing on the applicability of the ecological modernization approach in the Global South. However, as the issue is very complex, multi-disciplinary investigations are helpful to ensure comprehensive dynamics. A further study, for example, applying remote sensing image data and GIS techniques to illustrate the changes in the development and utilization of the area inside and outside the CEP dam is necessary, and presenting the results in more graphic form would be easier for many to understand. Moreover, in assessing the sustainability adaptation of the CEP in future, an evaluation index system can be constructed based on the conceptual framework of the space for feasible actions.

## Figures and Tables

**Table 1:** Selection of respondents for in-depth interview.

Participants	Area			Total
	Bagerhat	Khulna	Satkhira	
Upazila Agricultural Officer	1	1	1	3
Upazila Fisheries Officer	1	1	1	3
BWDB official	1	1	1	3
Key informant	1	1	1	3
Shrimp farmer	5	5	5	15
Total	9	9	9	27

## Data Availability

The data presented in this study are available on request from the corresponding author. The data are not publicly available due to some privacy issues.
